# Transcriptome analysis of callus from *Picea balfouriana*

**DOI:** 10.1186/1471-2164-15-553

**Published:** 2014-07-04

**Authors:** Qingfen Li, Shougong Zhang, Junhui Wang

**Affiliations:** State Key Laboratory of Forest Genetics and Tree Breeding, Research Institute of Forestry, Chinese Academy of Forestry, Number 1 of Dongxiaofu in Haidian District, Beijing, China

**Keywords:** *Picea balfouriana*, Somatic embryogenesis, Embryogenic tissue, Non-embryogenic tissue

## Abstract

**Background:**

*Picea likiangensis* var. *balfouriana* (Rehd. et Wils.) Hillier ex Slavin (also known as *Picea balfouriana*) is an ecologically and economically important conifer that grows rapidly under optimum conditions and produces high-quality wood. It has a wide geographic distribution and is prevalent in southwest and eastern regions of China. Under suboptimal conditions, *P. balfouriana* grows slowly, which restricts its cultivation. Somatic embryogenesis has been used in the mass propagation of commercial species. However, low initiation rates are a common problem and the mechanisms involved in the induction of somatic embryogenesis are not fully understood. To understand the molecular mechanisms regulating somatic embryogenesis in *P. balfouriana*, high-throughput RNA-seq technology was used to investigate the transcriptomes of embryogenic and non-embryogenic tissues from three *P. balfouriana* genotypes. We compared the genes expressed in these tissues to identify molecular markers with embryogenic potential.

**Results:**

A total of 55,078,846 nucleotide sequence reads were obtained for the embryogenic and non-embryogenic tissues of *P. balfouriana*, and 49.56% of them uniquely matched 22,295 (84.3%) of the 26,437 genes in the *Picea abies* genome database (Nature 497: 579-584, 2013). Differential gene expression analysis identified 1,418 differentially expressed genes (false discovery rate <0.0001; fold change ≥2) in the embryogenic tissues relative to the non-embryogenic tissues, including 431 significantly upregulated and 987 significantly downregulated genes. KEGG (Kyoto Encyclopedia of Genes and Genomes) pathway analysis revealed that the most significantly altered genes were involved in plant hormone signal transduction, metabolic pathways (starch and sucrose metabolism), and phenylalanine metabolism.

**Conclusions:**

We found that the initiation of embryogenic tissues affected gene expression in many KEGG pathways, but predominantly in plant hormone signal transduction, plant-pathogen interaction, and starch and sucrose metabolism. The changes in multiple pathways related to induction in the *P. balfouriana* embryogenic tissues described here, will contribute to a more comprehensive understanding of the mechanisms involved in the initiation of somatic embryogenesis. Additionally, we found that somatic embryogenesis receptor kinase (*SERK*), arabinogalactan proteins, and members of the WUS-related homeobox protein family may play important roles and could act as molecular markers in the early stage of somatic embryogenesis, as reported previously.

**Electronic supplementary material:**

The online version of this article (doi:10.1186/1471-2164-15-553) contains supplementary material, which is available to authorized users.

## Background

*Picea balfouriana* grows in a specific type of forest ecosystem in southwest China and is an eastern species on the Tibetan plateau. For rapid growth and production of high-quality wood, many plantations selected *P. balfouriana* as one of the main species for afforestation. Seedlings of *P. balfouriana* are produced primarily by sexual propagation; however, the seeds set late in the season, and the seedlings grow very slowly during the early phases.

Propagation by somatic embryogenesis (SE) is the primary technology used for many conifer biotechnology products, including the development of transgenic trees. The SE system for most spruce (*Picea* spp.) and some pine (*Pinus* spp.) species is sufficiently refined for commercial use; however, for many economically important conifer species, the initiation frequency of SE is insufficient for commercial applications. In recent years, much research has been focused on the development and optimization of protocols for the induction and maturation of somatic embryos of coniferous species [1]. The developmental stage of zygotic embryos, the contents of the growth or culture medium, the type and concentration of plant growth regulators, and culture condition factors such as pH, agar type, and nitrogen level can affect the generation of embryogenic tissue (ET). Therefore, understanding and balancing these factors is crucial for increasing the initiation rate of SE. A complete understanding of the genes involved in the development of ET and non-embryogenic tissue (NET) will improve the understanding of the initiation process in embryogenic tissue.

Cellular identity and function are determined by the transcriptome, i.e. the complete repertoire of expressed RNA transcripts. Transcriptome profiling is a powerful method that has been used widely to assess the relative importance of gene products in cells, tissues, organisms, or under different conditions. Next-generation sequencing (NGS) technology is a cutting-edge approach for high-throughput sequence determination, which has dramatically improved the efficiency and speed of gene discovery [2, 3]. For example, Illumina sequencing technology generates more than one billion bases of high-quality DNA sequence per run at less than 1% of the cost of capillary-based methods [4]. Thus, NGS has significantly accelerated and improved the sensitivity of gene-expression profiling, and is expected to boost collaborative and comparative genomics studies [5, 6]. Illumina sequencing of transcriptomes for organisms with completed genomes confirmed that the relatively short reads produced by this methodology can be assembled effectively and used for gene discovery and comparisons of gene expression profiles [7, 8]. Despite the obvious potential, NGS methods have not yet been used in *P. balfouriana*.

The high-throughput RNA-seq methodology involves whole-transcriptome shotgun sequencing, in which mRNA or cDNA is mechanically fragmented to produce overlapping short fragments that cover the entire transcriptome [9–11]. By mapping the RNA-seq reads to a reference genome, gene assignments and large-scale functional annotation of genes can be carried out. Further, compared with DNA microarray data, RNA-seq data can be used to detect and quantify RNAs that are expressed at very low levels [12]. Additionally, Gene Ontology (GO) analysis of the annotated genes can lead to a deeper understanding of the functions of the genes in cells, thereby providing sensitive and accurate profiling of the transcriptome and a description of gene function that resembles the biology of the cell [13].

Very few studies reporting the mechanism of initiation during SE have been published so far. The main objective of the present study was to identify differentially expressed genes in ETs and NETs, independent of genotype, through RNA-seq analysis and mapping to the reference *Picea abies* genome [14]. The results of this work may provide fundamental insights into the early SE in *P. balfouriana*.

## Results

### RNA-seq library sequencing

Six *P. balfouriana* RNA-seq libraries were produced and sequenced; namely, the ETs and NETs from three genotypes. In each library, 11.7–12.5 million clean reads were obtained after the low-quality reads were filtered out (Table 1). Among the clean reads, 8.4–10.9 million (71.24–87.31%) were mapped to known genes (Table 1).

**Table 1:** **Mapping of** ***Picea balfouriana*** **RNA-seq library reads to the** ***Picea abies*** **reference genome database**

Classification	ET Genotype1	NET Genotype 1	ET Genotype 2	NET Genotype 2	ET Genotype 3	NET Genotype 3
Raw reads	12,519,224	11,703,983	12,150,702	11,734,531	12,150,562	12,280,320
Base pairs	613,419,76	573,495,167	595,384,398	574,992,019	595,377,538	601,735,680
%	100.00	100.00	100.00	100.00	100.00	100.00
Mapped reads	5,713,055	5,044,017	5,590,502	4,359,900	5,519,889	5,456,021
%	45.63	43.10	46.01	37.15	45.43	44.43
Perfect match	3,923,702	3,401,759	3,865,351	2,947,585	3,870,249	3,674,461
%	31.34	29.06	31.81	25.12	31.85	29.92
<=2 bp mismatch	1,789,353	1,642,258	1,725,151	1,412,315	1,649,640	1,781,560
%	14.29	14.03	14.20	12.04	13.58	14.51
Unique match	5,374,869	47,721,812	5,262,180	4,081,332	5,214,938	5,117,424
%	42.93	40.34	43.31	34.78	42.92	41.67
Multi-position match	338,186	322,205	328,322	278,568	304,951	338,597
%	2.70	2.75	2.70	12.42	2.51	2.76
Unmapped reads	6,806,169	6,659,966	6,560,200	7,374,631	6,630,673	6,824,299
%	54.37	56.90	53.99	62.85	54.57	55.57

ET, embryogenic tissue; NET, non-embryogenic tissue.

Approximately 19,000 genes (short read mapped to reference genome) were detected in each library, accounting for more than 70% of the 26,437 genes in the *P. abies* reference genome database [14] (Table 2). Overall, 22,295 unique genes were expressed among the six libraries (Additional file 1), which gives a mapping coverage of 84.3%, conferring high confidence in the RNA-seq results. The mean coverage of all genes was above 60%, with the highest coverage at 99.96%. GO terms were assigned to the mapped genes and an enrichment analysis of the GO terms showed that intracellular organelle part, anion binding, and primary metabolic process were dominant in the cellular component, molecular function, and biological process categories, respectively. Some of the genes assigned primary metabolic process terms were associated with KEGG pathways involved in purine metabolism, RNA polymerase, pyrimidine metabolism, inositol phosphate metabolism and phenylalanine, and tyrosine and tryptophan biosynthesis (Additional file 2).

**Table 2:** **Genes found in** ***Picea balfouriana*** **that uniquely match genes in the** ***Picea abies*** **reference genome**

	ET Genotype 1	NET Genotype 1	ET Genotype 2	NET Genotype 2	ET Genotype 3	NET Genotype 3
Uniquely matched genes	18865	19256	19432	19143	19116	19287
Percentage	71.36%	72.84%	73.50%	72.41%	72.31%	72.95%

ET, embryogenic tissue; NET, non-embrygeonic tissue.

### Uniform genes with consistent fold-changes in ETs and NETs from the three genotypes

After two-factor analysis of variance, 4,734 genes with significantly different effects for “embryonic *vs*. non-embryonic” (P < 0.05) and insignificant effects for “genotype” (P > 0.05) were selected (Additional file 3). Then, the selected genes were analyzed using DESeq(version 2.14) to test for differentially expressed genes (DEGs) in ETs and NETs, independent of genotype (false discovery rate (FDR) <0.001, fold change ≥2). Ultimately, 1,418 DEGs between ET and NET were identified, among which 431 were upregulated and 987 were downregulated (Additional file 4). Some of the DEGs were associated with SE (germin-like proteins, abscisic acid receptor, cytochrome P450, and chitinases). About one-quarter of the genes were upregulated by ≥2-fold, and about 1,000 genes were downregulated by ≥2-fold.

Oxidoreductase activity, cell wall, and apoplast were the most enriched GO terms, while biosynthesis of secondary metabolites, metabolic pathways, pentose and glucuronate interconversion, starch and sucrose metabolism, phenylpropanoid biosynthesis, plant hormone signal transduction, flavonoid biosynthesis, phenylalanine metabolism, ether lipid metabolism, and propanoate metabolism were the most overrepresented KEGG pathways among genes related to embryogenic competence (Additional file 5). These findings will be very important for further understanding the intracellular signaling mechanisms of early SE in conifers.

### Expression of housekeeping genes in the different tissue samples of *P. balfouriana*

The NormFinder algorithm uses a model-based approach to estimate alterations in the expression of housekeeping genes [15]. The algorithm also estimates variations across subgroups and avoids the artificial selection of co-regulated genes. The results of the NormFinder analysis of the genes detected in the six libraries show that WS0109_C05 [TAIR: At1g29260] and 18S rRNA were predicted to be the most stable housekeeping genes (Table 3).

**Table 3:** **Ranking of** ***Picea balfouriana*** **candidate reference genes generated by NormFinder**

Gene name	Stability value	Standard error
WS00912.B21_N13	0.403	0.268
WS0109_C05	0.282	0.311
18S rRNA	0.923	0.324
Tubulin	0.779	0.295

BestKeeper measures stability using a pair-wise correlation analysis of all pairs of candidate genes and calculates the geometric mean of the best candidates [16, 17]. A preliminary analysis based on the inspection of raw Ct values estimated the variation of four housekeeping genes (WS00912.B21_N13, WS0109_C05, 18S rRNA, and tubulin) to be compatible with an overall stability in gene expression (Table 4). The standard deviation values were <1. The four housekeeping genes were used to calculate the BestKeeper index. BestKeeper allows a comparative analysis across internal reference genes by estimating correlations in the expression levels between all the possible candidates. Highly correlated control genes are combined into an index, and the pair-wise correlation between genes and the correlation between each gene and the index are also calculated. The results describe the consistency between the index and each internal reference gene. The four control genes tested in our analysis correlated well one with one another and with the NormFinder index (Table 3). The best correlation between one internal reference gene and the BestKeeper index was for WS0109_C05 (*r* = 0.947). The statistically significant correlation for WS0109_C05 with the BestKeeper index appeared to be consistent with the good performance of this housekeeping gene as assessed by NormFinder.

**Table 4:** **Statistical output from the BestKeeper analysis for the** ***Picea balfouriana*** **candidate reference genes**

	WS00912.B21_N13	WS0109_C05	18SrRNA	Tubulin
*N*	6	6	6	6
G Mean [Ct]	28.03	27.57	13.12	24.37
A Mean [Ct]	28.06	27.58	13.13	24.4
Min [Ct]	26.21	26.38	12.33	22.62
Max [Ct]	29.78	28.79	13.81	25.68
SD [±Ct]	0.85	0.58	0.56	1.06
CV [% Ct]	3.02	2.09	4.26	4.35
Coeff. of corr. [*r*]	0.85	0.947	-0.014	0.605
*P*-value	0.032	0.004*	0.978	0.203

*N*, number of samples; G Mean [Ct], geometric mean of the Ct; A Mean [Ct], arithmetic mean of the Ct; Min [Ct] and Max [Ct], extreme values of the Ct; SD [±Ct], standard deviation of the Ct; CV [% Ct], coefficient of variance expressed as percentage on the Ct level. Coeff. of corr. [*r*], coefficient of correlation. *Indicates the best correlation between the control genes and the BestKeeper index.

### Quantitative real-time reverse transcription-polymerase chain reaction (qRT- PCR) analysis

The peroxisomal targeting signal receptor WS0109_C05 displayed constitutive expression in all the analyses performed using the *P. balfouriana* RNA-seq data; therefore, it was used as an internal control for the normalization of gene expression levels (ΔCT). Total RNA from the same six tissue samples that were used for the RNA-seq analysis were used as templates for the qRT-PCR analysis (Table 5). For most of the genes, the transcript fold-ratios determined by qRT-PCR were approximately the same as those estimated from the RNA-seq data, indicating the reliability of the RNA-seq in *P. balfouriana*.

**Table 5:** **Comparison of RNA-seq data with expression data from qRT-PCR**

NCBI gene ID	UniProt_ID	Description	Method	Expression level in ET	Expression level in NET	Fold change
840475	Swiss-Prot:Q9LP24	Leucine-rich repeat receptor-like protein kinase	RNA-seq	Log-cpm in ET	Log-cpm in NET	log2 ratio (ET/NET)
				605.5	3640.6	2.6
			qRT-PCR	ET(ΔCT)	NET(ΔCT)	2^-ΔΔCT^
				9.6	4.6	2.1
817958	Swiss-Prot:Q6X7J4	WUSCHEL-related homeobox 9	RNA-seq	Log-cpm in ET	Log-cpm in NET	log2 ratio (ET/NET)
				155.7412756	0.353625	8.8
			qRT-PCR	ET(ΔCT)	NET(ΔCT)	2^-ΔΔCT^
				3.0	1.3	3.4
831649	Swiss-Prot:Q8GY25	WUSCHEL-related homeobox 12	RNA-seq	Log-cpm in ET	Log-cpm in NET	log2 ratio (ET/NET)
				136.2	1.8	6.3
			qRT-PCR	ET(ΔCT)	NET(ΔCT)	2^-ΔΔCT^
				0.7	0.1	1.5

Three biological repeats were reverse transcribed and amplified independently. The raw threshold cycle (Ct) values were normalized against the WS0109_C05 standard to generate the normalized ΔCt values that were used to calculate the fold-change in expression in embryonic tissue (ET) and non-embryonic tissue (NET) of the three genotypes. Log-cpm, log-counts per million.

## Discussion

Most early studies on the initiation mechanism of SE in *P. balfouriana* focused mainly on different genes between ET and NET from just one cell line. It has been suggested that genotype may have an important effect on initiation of ET. In this study, we demonstrated that many genes were influenced by genotype, so we filtered them out and selected the genes that were related only to embryogenic ability. Six RNA-seq libraries were created to analyze the DEGs in ET and NET, yielding a total of 22,295 genes. Among them, 9,988 (44.8%) and 13,135 (58.9%) were enriched in GO terms and KEGG pathways, respectively. Our transcriptome and gene expression profiling data will greatly enrich the current knowledge for the genus *Picea*, and will contribute to the database for conifer species. These data will also help promote research on the identification of novel genes related to embryogenic competence.

Several genes involved in the response to phytohormone and hormone-mediated signaling pathways in ETs were strongly upregulated (Additional file 4); for example, the genes that encode auxin-induced proteins. The initiation of embryogenesis in somatic tissues requires auxins, especially in conifer species. Exogenous hormones and culture-related stress has been reported to play important roles in triggering SE, which presumably results from the expression of genes involved in cell division, cell polarization, and the regulation of signal transduction pathways. Several auxin-inducible genes that are involved in cellular changes, such as activation of cell division, have been cloned [18, 19]. Bögre et al. [20] used auxin-responsive genes as molecular markers to distinguish embryogenic from non-embryogenic genotypes of *Medicago sativa*. Our results indicated that most of the DEGs were involved in plant-pathogen interaction, metabolic pathways (starch and sucrose metabolism), and plant hormone signal transduction (Additional file 5) and, therefore, they may play essential roles in the initiation of SE.

A number of genes encoding membrane-localized, leucine-rich, repeat receptor-like kinases, such as IMK2 [Swiss-Prot:Q9SCT4], were among the DEGs. The somatic embryogenesis receptor kinase (*SERK*) gene encodes a leucine-rich repeat receptor-like kinase that plays an important role in plant signaling pathways. The *SERK* gene was first identified in carrot (*Daucus carota*) suspension cultures where it was specifically expressed in cells that developed into somatic embryos [21]. *SERK* has been linked to SE in a number of species, including *Dactylis glomerata* [22], *Arabidopsis thaliana* [23], *Medicago truncatula* [24], sunflower (*Helianthus annuus*) [25], *Ocotea catharinensis* [26], *Citrus unshiu* [27], and *Theobroma cacao* [28]. There is evidence that *SERK* genes are required for embryo initiation in embryogenic cells [21, 23, 29]. In *D. glomerata*, and *A. thaliana*, *SERK*s are characteristic markers of embryogenic cell cultures and somatic embryos [21–23].

Several arabinogalactan proteins (AGPs) were upregulated in ETs compared with NETs, such as FLA7[Swiss-Prot:Q9SJ81]. AGPs have been implicated in five fundamental cellular processes: cell proliferation, cell expansion, cell differentiation, programmed cell death, and cell-cell communication [30]. The involvement of AGPs in SE has been studied previously [31–37], and the role of AGPs in the establishment of embryogenic cell cultures and the influence of AGPs on cell development have also been examined. The presence of AGPs in seeds was found to increase the number of pro-embryogenic masses and the embryogenic potential [38–41]. Filonova et al. [42] reported the importance of intercellular communication for the acquisition of embryogenic competence, and observed that separation of Norway spruce cells by fractionation of suspension cultures inhibited the embryogenic process. AGPs restore differentiation potential after cell wall removal, and this restoration was reported to be more efficient with chitinase-cleaved forms of AGPs [36]. Based on these observations, we propose that complex interactions between cells and substances secreted in the medium of embryogenic cultures are essential to establish and maintain embryogenic competence in culture.

The qRT-PCR results confirmed the observed upregulation in ETs of two genes belonging to the WUS-related homeobox (*WOX*) family. Previous studies have reported the expression dynamics of *WOX* and shown that it is highly expressed during early somatic embryo development, but declines as the embryo matures [43–45]. Haecker et al. [43] found that *WOX8* and *WOX9* were expressed in *A. thaliana* during embryo development from the very early stages and throughout development. Several *WOX* genes, including *WOX2*, were reported to constitute potential markers of cell fate during early embryogenesis, suggesting that they might have important functions in early embryonic patterning [44]. Palovaara et al. [45] found that *WOX2* played a fundamental role in early somatic embryo development in *P. abies*, possibly related to the regulation of cell division and/or differentiation in the embryos. It was also proposed that *WOX2* could be used as a molecular marker of embryogenic potential, but was not necessary for the regenerative capacity of cell lines [45]. Klimaszewska et al. [46] reported that *WOX2* was expressed exclusively in the early stages of SE, and might be useful as a marker of embryogenic potential. *WOX3*, *WOX8A*, and *WOX8B* were all significantly expressed in proembryogenic masses in *P. abies* rather than in somatic embryos [47].

## Conclusions

Although ET and NET can be discriminated by visual inspection, and molecular markers of embryogenic potential are useful, bioinformatics tools provide a powerful approach to identify DEGs in ETs and NETs. Here, we found gene expression patterns in ETs from *P. balfouriana* that identified changes in multiple pathways related to early SE, such as plant hormone signal transduction, metabolic pathways (starch and sucrose metabolism), and phenylpropanoid biosynthesis. If these pathways could be modified to induce ETs with higher initiation rates that have competence to form normal somatic embryos they could be used to increase the initiation frequency of SE for commercial applications.

A large number of candidate genes, such as those that encode heat shock proteins and glutathione S-transferases, showed significant different expression in the ET libraries compared with their expression in the NET libraries; however, these results require further verification and characterization. Although the molecular functions of individual *P. balfouriana* genes and the associated signal transduction pathways often remained largely unknown, the RNA-seq analysis has provided valuable information about the induction of SE in *P. balfouriana*, which could promote further investigations into the detailed physiological initiation mechanisms of SE. This analysis represents a starting point for detailed functional studies; however, further experiments are required to expand on these results and to define the complex interaction networks and molecular mechanisms responsible for the induction of SE in *P. balfouriana*.

## Methods

### Plant material

**Figure 1:**
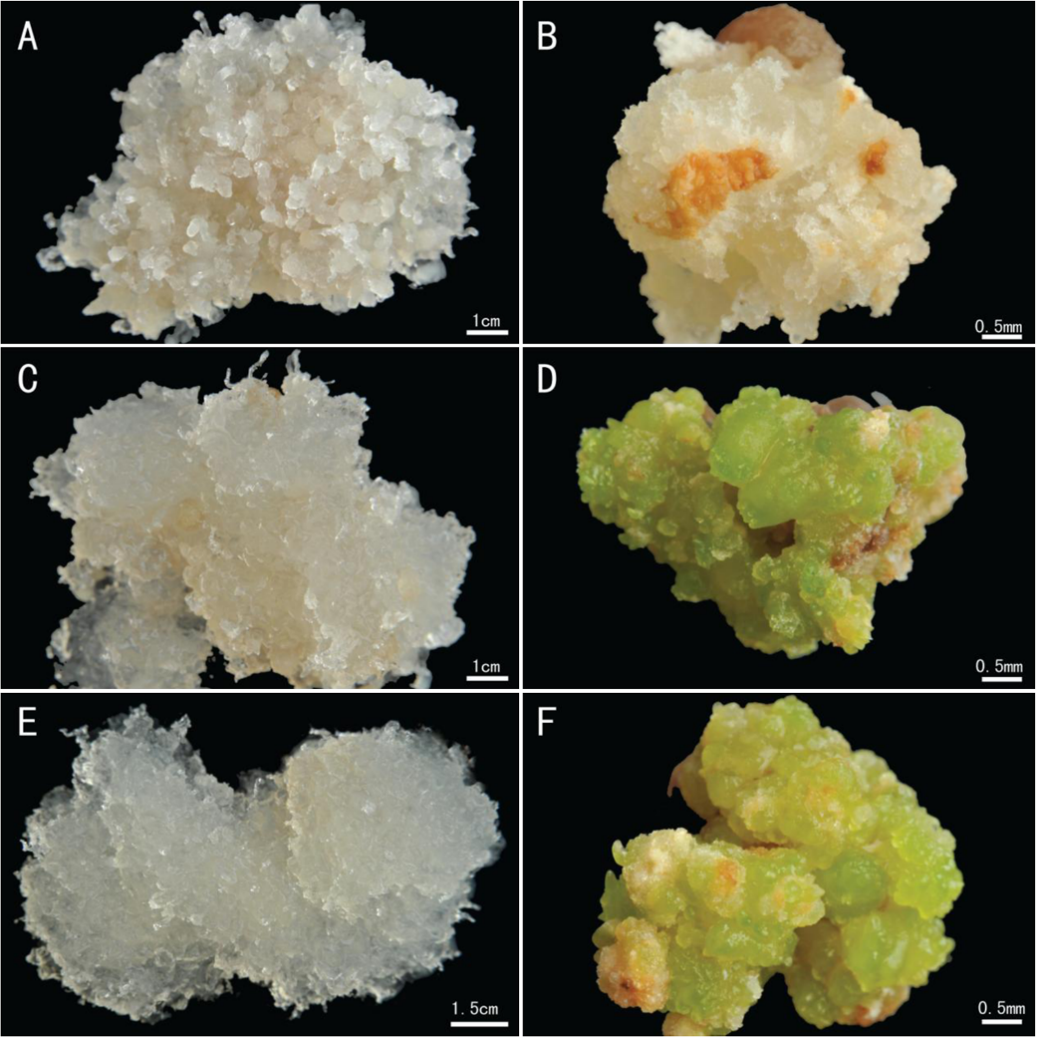
**Embryonic tissue (ET) and non-embryonic tissue (NET) of three** ***Picea balfouriana*** **genotypes.** **(A)** and **(D)** ET (bar = 1.0 cm) and NET (bar = 1.5 cm) of genotype 1, respectively; **(B)** and **(E)** ET (bar = 1.0 cm) and NET (bar = 1.5 cm) of genotype 2, respectively; **(C)** and **(F)** ET (bar = 1.0 cm) and NET (bar = 2.0 cm) of genotype 3, respectively.

ETs and NETs were collected from three genotypes of *P. balfouriana* (Figure 1), and initiated and maintained on 1/2 LM basal medium [48], supplemented with 2.2 mg · L^-1^ 2,4-dichlorophenoxyacetic acid (2,4-D), 1.1 mg · L^-1^ BAP, 0.2 mg · L^-1^ KT, 1% sucrose, 1000 mg · L^-1^ casein hydrolysate, 1% sucrose, 500 mg · L^-1^ glutamine, and 0.2% gellan gum (Sigma, St. Louis, MO, USA).

### RNA isolation

Total RNA was extracted immediately from fresh ET and NET samples, TRIZOL® reagent (Invitrogen, Carlsbad, CA, USA) was used to extract total RNA from 50 to 100 mg of tissue, according to the manufacturer’s instructions. The RNA samples were incubated for 30 min at 37°C with 10 U of DNaseI (Takara, Dalian, China) to remove residual genomic DNA. The quality and quantity of the purified RNA were determined by measuring the absorbance at 260 nm and 280 nm (A260/A280) using a Nano-drop® ND-1000 spectrophotometer (LabTech, Holliston, MA, USA). The samples had an average RNA integrity number (RIN) of 8.9 based on a lab-on-chip analysis using a 2100 Bioanalyzer (Agilent Technologies, Santa Clara, CA, USA).

### RNA-seq library preparation and sequencing

After extracting total RNA from the six samples, oligo (dT) magnetic beads were used to enrich the mRNA by removing rRNA. Fragmentation buffer was used to break the mRNA into short fragments (about 200-bp long), Then a random hexamer-primer was used to generate first-strand cDNA using the mRNA fragments as templates. Buffer, dNTPs, RNase H, and DNA polymerase I were added to synthesize the second-strand cDNA. A QiaQuick PCR extraction kit (Qiagen, Hilden, Germany) was used to purify the double-strand cDNA, which was then washed with EB buffer for end repair and poly(A) addition. Finally, sequencing adaptors were ligated onto the fragments. Agarose gel electrophoresis was used to purify the fragments, which were then enriched by PCR amplification. The library products were sequenced using a Illumina HiSeqTM 2000 system (Illumina, San Diego, USA).

### Analysis and mapping of RNA-seq reads

The sequenced raw data were filtered to remove low-quality tags (reads with unknown nucleotides “N”), empty reads (no read sequence between the adaptors), and reads with only one copy number (reads that might have resulted from sequencing errors). SOAPaligner/soap2 (version2.21) [49] was used to align the remaining clean reads to the sequences in the *P. abies* reference genome database, allowing up to two base mismatches. At most, one continuous gap or two mismatches were allowed in the high-quality part of a read. The mapped clean reads were designated as unambiguous clean reads. For two-factor analysis of variance, the number of unambiguous clean reads for each gene was calculated and normalized to log-counts per million using the latest version of the limma package in R [50]. The RNA-seq data consisted of a matrix of read counts *r*_*gi*_, for RNA samples *i* = 1 to n, and genes *g* = 1 to G. If *R*_*i*_ for the total number of mapped reads for sample i is defined as , then the log-counts per million (log-cpm) value for each count can be calculated as 

The counts were offset away from zero by adding 0.5 to avoid taking the log of zero, and to reduce the variability of log-cpm for low expression genes. The library size was offset by 1 to ensure that (*r*_*gi*_ + 0.5)/(*R*_*i*_ + 1) was always less than 1 and greater than zero.

### Differentially expressed genes between the ET and NET libraries

Two-factor analysis of variance for each gene, using two factors, “embryonic *vs*. non-embryonic” and “genotype”, was performed in R (version 2.15.) to detect genes that were differentially expressed between the ET and NET libraries, and not affected by genotype. DESeq was also used to detect the DEGs between the ETs and NETs [51]. Finally, genes with significant effects from “embryonic vs. non-embryonic” (P < 0.05) and insignificant effects from “genotype” (P > 0.05) in two-factor analysis of variance were selected from the significantly changed genes detected by DESeq and considered as the DEGs between the ET and NET libraries. The FDRs of these DEGs were less than 0.001 and the absolute values of the log_2_ ratio were greater than 1 [52]. Briefly, if *R* DEGs are selected and *S* genes are genuinely differentially expressed while *V* genes are false positives, and if the error ratio (Q = *V*/*R*) is required to remain below a cutoff (e.g. 0.001), then the FDR can be calculated according to the Benjamini and Hochberg algorithm [52] as: *FDR* = *E*(*Q*) = *E*{*V*/(*V* + *S*)} = *E*(*V*/*R*).

In this study, a FDR of ≤0.001 was used.

All the genes, including the DEGs, were used for the GO and KEGG ontology (KO) enrichment analyses. For the GO analysis, a corrected P-value of <0.05 was used as the threshold to determine significant enrichment of the gene sets. For KO enrichment analysis, a Q-value ≤0.05 was used as the threshold to determine significant enrichment of the gene sets.

### Validation of DEGs by qRT-PCR

Four reference housekeeping genes were selected for *P. balfouriana* and forward and reverse primers were designed as follows: WS00912.B21_N13 (5′-GTCGTGTGGATTGTCTCTGC-3′; 5′-ATGTATTCGAAGAGGAGGAATG-3′), WS0109_C05 (5′-AACTGTCATTGGAGTCCTGTAGG-3′; 5′-TGAGGGTTTGGGT AGTGAGA-3′), tubulin (PaTubF: 5′-CGTTACCTGCTGCCTGAG-3′, PaTubR: 5′-G CTCTGTATTGCTGTGAACC-3′), and 18S rRNA (Pa18SF:5′-CGGCGGATG TTGCTCTAAG-3′; Pa18SR:5′-TCTGTCAATCCTTACTATGTCTGG-3′). Those gene has been used as a reference in other studies [53, 54]. The most stable gene (WS0109_C05) was selected as the reference gene. Details of the qRT-PCR analysis of spruce transcripts has been described previously [55]. In brief, total RNA (18 μg) from each sample was treated with DNase I (Invitrogen). The absence of DNA in the treated RNA samples (10 ng) was confirmed by PCR using the WS0109_C05 primers. Next, RNA (three reactions of 4 μg each per sample) was reverse transcribed, and cDNA (10 ng) was analyzed by PCR in a total volume of 20 μL, in the presence of 10 μL DyNAmo SYBR Green Mastermix (FinnZymes, Espoo, Finland) and 0.3 μM each of a forward and a reverse primers. Primers of 20–24 nucleotides in length (usually located in the 3′-untranslated region) with a *T*_*m*_ of 60°C were designed to amplify a gene-specific, 160–200 bp fragment of the target cDNA. Reactions were carried out in triplicate in an ABI7900HT RT-PCR machine (ABI, Foster City, USA)with an initial step of 3 min at 95°C, followed by 40 cycles of 3 s at 95°C and 20 s at 60-63°C. Each cycle was followed by a data-acquisition step. After the last cycle and a final 10-min extension at 72°C, a melting curve was measured from 60 to 95°C with measurements every 0.2°C and holding for 1 s. Fluorescence was determined following each annealing and extension phase.

### Availability of supporting data

The RNA-seq data supporting the results of this article are available at the NCBI under BioProject (http://www.ncbi.nlm.nih.gov/bioproject/211928) with accession number PRJNA211928.

## Electronic supplementary material

Additional file 1: **Details of the 22,295 genes identified in six libraries of** ***Picea balfouriana.*** (XLS 8 MB)

Additional file 2: **Enriched GO terms and KEGG pathways for the 22,295 genes identified in six libraries of** ***Picea balfouriana.*** (XLS 36 KB)

Additional file 3: **Genes selected from two-factor analysis of variance as differentially expressed between the ET and NET libraries.** (XLS 705 KB)

Additional file 4: **Differentially expressed genes between the ET and NET libraries confirmed by DESeq.** (XLS 470 KB)

Additional file 5: **Enriched GO terms and KEGG pathways for the differentially expressed genes between the ET and NET libraries.** (XLS 48 KB)

## Abbreviations

AGP: Arabinogalactan proteins
ET: Embryogenic tissue
NET: Non-embryogenic tissue
FDR: False-discovery rate
*Picea balfouriana*: *Picea likiangensis* var. *balfouriana* (Rehd. et Wils.) Hillier ex Slavin
*SERK*: Somatic embryogenesis receptor-like kinase.
